# Self-reported Subjective Effects of Analytically Confirmed New Psychoactive Substances Consumed by e-Psychonauts: Protocol for a Longitudinal Study Using a New Internet-Based Methodology

**DOI:** 10.2196/24433

**Published:** 2021-07-02

**Authors:** Marc Grifell, Guillem Mir Fuster, Mireia Ventura Vilamala, Liliana Galindo Guarín, Xoán Carbón Mallol, Carl L Hart, Víctor Pérez Sola, Francesc Colom Victoriano

**Affiliations:** 1 Mental Health Research Group Hospital del Mar Medical Research Institute Barcelona Spain; 2 Department of Psychiatry and Legal Medicine Universitat Autònoma de Barcelona Barcelona Spain; 3 Laboratory of Neuropsychopharmacology Department of psychology Columbia University New York, NY United States; 4 Energy Control Asociació Benestar i Desenvolupament Barcelona Spain; 5 Institute of Neuropsychiatry and Addiction Parc de Salut Mar Premia De Mar Spain; 6 Cambridgeshire and Peterborough NHS Foundation Trust Cambridge United Kingdom; 7 Department of Psychiatry University of Cambridge Cambridge United Kingdom; 8 Division on Substance Abuse New York State Psychiatric Institute and Department of Psychiatry, College of Physicians and Surgeons Columbia University New York, NY United States; 9 Centro de Investigación Biomédica en Red de Salud Mental Madrid Spain; 10 Departament of Basic, Evolutive and Education Psychology Universitat Autònoma de Barcelona Barcelona Spain

**Keywords:** psychotropic, psychoactive, psychonautic, longitudinal, observational, pharmacology, psychopharmacology, subjective effects, sentinel, mental health, public health, internet, eHealth, cathinones, drugs of abuse, psychedelics, mobile phone, smart phone, online recruitment, online forums

## Abstract

**Background:**

During the last few years, the continuous emergence of new psychoactive substances (NPS) has become an important public health challenge. The use of NPS has been rising in two different ways: buying and consuming NPS knowingly and the presence of NPS in traditional drugs as adulterants. The rise of NPS use is increasing the number of different substances in the market to an extent impossible to study with current scientific methodologies. This has caused a remarkable absence of necessary information about newer drug effects on people who use drugs, mental health professionals, and policy makers. Current scientific methodologies have failed to provide enough data in the timeframe when critical decisions must be made, being not only too slow but also too square. Last but not least, they dramatically lack the high resolution of phenomenological details.

**Objective:**

This study aims to characterize a population of e-psychonauts and the subjective effects of the NPS they used during the study period using a new, internet-based, fast, and inexpensive methodology. This will allow bridging an evidence gap between online surveys, which do not provide substance confirmation, and clinical trials, which are too slow and expensive to keep up with the new substances appearing every week.

**Methods:**

To cover this purpose, we designed a highly personalized, observational longitudinal study methodology. Participants will be recruited from online communities of people who use NPS, and they will be followed online by means of a continuous objective and qualitative evaluation lasting for at least 1 year. In addition, participants will send samples of the substances they intend to use during that period, so they can be analyzed and matched with the effects they report on the questionnaires.

**Results:**

The research protocol was approved by the Institutional Review Board of the Hospital del Mar Research Institute on December 11, 2018. Data collection started in August 2019 and was still ongoing when the protocol was submitted (September 2020). The first data collection period of the study ended in October 2020. Data analysis began in November 2020, and it is still ongoing. The authors expect to submit the first results for publication by the end of 2021. A preliminary analysis was conducted when the manuscript was submitted and was reviewed after it was accepted in February 2021.

**Conclusions:**

It is possible to conduct an institutional review board–approved study using this new methodology and collect the expected data. However, the meaning and usefulness of these data are still unknown.

**International Registered Report Identifier (IRRID):**

DERR1-10.2196/24433

## Introduction

### Importance of New Psychoactive Substances

To date, new psychoactive substances (NPS) still represent a very important challenge to legislate, monitor, study, and develop health interventions. The understanding of use patterns remains poor, with most information being based on populations and settings where problems have already occurred [1].

The ever-increasing number of psychoactive substances used nowadays represents a new challenge for psychiatry, as the pharmacodynamics and pharmacokinetics of many NPS are not yet thoroughly understood [2]. In addition, NPS consumption rarely occurs in isolation from other habits but, on the contrary, is placed within a kaleidoscopic range of poly drug use trajectories. There seems to be no differential risk for NPS use compared with the use of traditional psychoactive substances such as alcohol, cannabis, or cocaine [1].

This new phenomenon represents an unprecedented challenge in the field of drug use as well as a fast-growing problem from social, cultural, legal, and political perspectives [3].

### NPS: Definition

NPS are substances of abuse, either in a pure form or a preparation, that are not controlled by the 1961 Single Convention on Narcotic Drugs or the 1971 Convention on Psychotropic Substances but which may pose a public health threat. It is important to note that different authors have previously referred to them as *designer drugs*, *legal highs*, *herbal highs*, *bath salts* and *research chemicals*. Moreover, the term *new* does not necessarily refer to new inventions but to substances that have recently emerged on the market [4]. Hence, *new* can include a failed pharmaceutical or an old patent that has been *rediscovered* for *recreational* substance [2].

Another distinction being made is between NPS and emerging psychoactive substances, where the latter term captures all NPS as well as drugs that may not be newly invented but have recently experienced a resurgence of, or increase in, use [2]. However, to simplify this work, only the term NPS will be used, also including all emerging psychoactive substances. Most NPS are the result of minor changes to the molecular structure of well-known legal or illegal drugs, such as opioids, ecstasy, or stimulants [5].

Between 2009 and 2017, 803 NPS were reported in 111 countries or territories [2,6]. In the European Union, by the end of 2017, the number of NPS was over 670, of which 632 were notified after 2004 [2,7]. However, evidence suggests that the NPS scenario could be much larger than that formally identified by international agencies. In a recent publication, Schifano et al [2] used a web search engine to identify NPS discussed online by NPS enthusiasts. Using this methodology, they identified a few thousand NPS, a number which is about 4-fold higher than the figures suggested by European and international drug agencies.

There is an ongoing debate on the scale of challenges posed by NPS, as the evidence on the prevalence of NPS use is scarce. For example, general population surveys suggest that the prevalence of NPS use is relatively low, with the best estimates found in the scientific literature being between 1% and 2% in the United Kingdom. However, the speed of technological innovation and the ease of synthesizing NPS present substantial challenges to regulatory authorities, researchers, and clinicians [5,8].

### NPS: Challenges

NPS may now pose a big challenge due to several factors:

1. NPS consist of several different classes of substances, which vary in their psychological and physiological effects. Treatment is often difficult because of the young age of most users and the possibility of concurrent polysubstance use. The pattern of use is often intermittent in social settings, so it may be perceived as less of a problem [9].
2. NPS appear into—and sometimes disappear from—the market very quickly, and as such, they are not significantly impacted by regulatory efforts. Currently, new substances are identified in Europe at a rate of one or more per week [10]. Several key studies have shown the continued use and popularity of mephedrone, a popular NPS, among specific drug-using populations after it was brought under control. The scheduling of new substances could even increase the speed at which manufacturers innovate, to bypass the law [11].
3. NPS are mainly distributed through the internet in a transnational market without solid information about their effects and risks [11]. During recent years, the widespread availability of internet access has led to a gradual, although only partial, shift from a *street* to a *web* market [12]. The increased web-based distribution has been seen in both the surface web and *dark net* [13].
4. NPS can substitute traditional drugs in times when their availability is restricted [8]. This could be problematic, as this substitution happens both by introducing new substances in the market as well as by selling NPS as traditional drugs, exposing large populations, unknowingly, to the effects of a new unstudied substance without previous experiences. This is especially dangerous, combined with the rapid turnover of NPS, as they change before we can obtain research data using conventional methodologies [5].
5. There is a concerted effort to grapple with the challenges of researching NPS, as traditional methodologies are too slow and expensive to generate relevant and timely data on the effects of NPS [5].
6. Clinicians are not usually able to identify a potential NPS user, and NPS usually produce negative results to traditional drug tests, which are designed to assess a very limited number of traditional substances [6]. On the other hand, NPS users rarely search for professional help linked exclusively to this problem, and clinicians are not trained to screen or identify NPS use.

### What Has Been Done and What Is Needed in NPS Research?

Despite the high number of publications about NPS during the last 20 years, especially after a sharp increase in 2010, there are still concerning *gaps in our understanding* of the phenomena. From the evidence map about the NPS research performed by Mdege et al [8], 2 things appear quite striking:

- First, most of the studies were performed in a general hospital population (118/294, 40.1%) or specialist settings (24/134, 18.2%), with relatively *low rates of studies coming from the internet population (*5/59, 8%). In addition, these studies mainly reported severe intoxication or other acute NPS-related problems.
- Second, the most frequent study design reported in the indexed peer-reviewed literature was case series and/or reports (n=367), followed by the literature review (n=243), the survey (n=130), and the secondary quantitative data analysis (n=99), with only 13 existing randomized controlled trials, 6 prospective cohort studies, and 1 case-control study [5,8].

There are also some *specific limitations* to the research performed till date. Although the most robust and representative data on NPS use are for mephedrone (surveys have been conducted in the United Kingdom since 2010), Mdege et al [8] acknowledge important limitations to this most robust research. For example, although participants may report using a substance, the names of NPS are sometimes used interchangeably, and *there is no analytical confirmation* of the true compound that was taken. Therefore, there is inherent uncertainty in the reported use of a particular NPS.

The same authors also reported that sentinel populations are likely to be at a greater risk of NPS use. However, it remains mostly limited to attendees of nightclubs where different sexual orientations are accepted. Other authors have also remarked that only a handful of studies have moved beyond prevalence to explore subjective user experiences and motivations [11].

Currently, the potential data sources that can provide some information on the acute effects of NPS consumption are as follows: (1) user self-reports on internet discussion forums, (2) surveys answered by users, and (3) fatal and nonfatal case reports.

Self-reports and surveys are mostly based on self-reported use rather than the analytical confirmation of the substances used. In contrast, case reports are usually generated from hospital settings in the context of an intoxication or overdose with multiple substances involved, so there is analytical confirmation of the substance but no self-reported effects. Unsurprisingly, the literature is dominated by studies investigating the problems associated with NPS (773/995, 77.7% of records). Therefore, caution is required when interpreting these data because of the following limitations:

- Users will report what they believe they have used, rather than whatever substance is actually taken [10].
- Intoxications with multiple substances in hospital settings do not target the information on psychopharmacological effects of a particular NPS [5,8].

In their empirical and conceptual review to produce research recommendations, Mdege et al [8] provide the following advice for research, among others:

1. The need to be aware of innovation opportunities, such as testing emerging NPS brands online as they become available.
2. Using cohort study designs to better understand the determinants of NPS use and related physical and mental health, psychosocial problems, and how patterns of involvement and consequences change over time.
3. What are the prevalence and patterns of NPS use in the general UK population and do they differ between subgroups of the population?
4. Are there sentinel populations capable of being monitored to provide early warnings of new trends?
5. Which acute intoxication problems are associated with NPS use?
6. Which promising approaches are currently available or can be made available in the United Kingdom for intervening with NPS use?

Finally, they concluded that there is a need for a major research effort to be directed at NPS, which should address NPS together with other forms of licit and illicit drug use [5,8].

### The e-Psychonaut Population

Both the limitations and recommendations stated above lead to the *necessity of conducting a longitudinal study in a specific and potential sentinel population*, such as internet NPS–consuming communities. This would allow for early assessments of the effects of recently emerged drugs and to study the patterns of consumption, harm reduction strategies, and long-term drug-related problems.

Available data on sentinel populations are growing. For example, several studies of attendees of gay-friendly night clubs suggest that the trend in reduction of mephedrone witnessed nationally may also occur in this subgroup. However, the study of this sentinel population has failed to predict future harms and trends in the global NPS market [5].

Conversely, data on another sentinel population, namely, e-psychonauts, have been able to predict future NPS-related harms occurring in more general settings [2].

In fact, the sentinel population of *e-psychonauts* has been considered by several authors as potentially useful in identifying NPS availability, market, and diffusion in advance. This population is believed to be responsible for shaping and influencing the drug scenarios of the future [13]. In addition, Corazza et al [3] provided evidence supporting the claim that the online NPS scenario predicts the real-life NPS scenario.

The term psychonaut was first described by Newcombe [14] as an adult user of psychoactive drugs who takes these substances in *normal, everyday settings* with the intention of subjectively exploring their effects.

Some years later, O’Brien et al [11] coined the term *cyber-psychonauts* to refer to their sample composed predominantly of NPS consumers. Cyber-psychonauts are further defined by their commitment to harm reduction, to using NPS safely and responsibly, and to purchasing chemicals online [8].

Tackett-Gibson [15] also documents the existence of online communities populated by self-defined *experts* in using NPS, providing a contrasting narrative around drug use and risk to that established by the scientific community. A brief perusal of relevant websites confirms the existence of a great number of NPS-related discussion threads, suggesting the existence of an online community of more discerning NPS users [15].

Orsolini et al [13] also refer to this population in their more recent study, identifying *educated and informed* users within web-based drug forum communities, who can provide reliable information on psychoactive compounds. They refer to these users as *e-psychonauts*, providing the best characterization of the population to date [13]. The e-psychonauts appear to be mainly young and unmarried White males, presenting good or excellent employment conditions and with a set of key skills, including awareness to their inner *soul*; high standards of knowledge about drugs’ chemical and pharmacological issues; and high levels of both technology-related skills. They are meant to have a wide vocabulary to define their own *on drug* experiences in the most subtle and precise way possible.

Among this population, the frequency of NPS use is high, with one-third of the participants reporting its use in the last week. They view themselves as knowledgeable consumers who use the internet to accumulate information about NPS and share their own experiences, informing fellow users of potential harms. However, other studies [16] reported possible stimulant dependence (3 or more dependence symptoms) in 30% of mephedrone users. Mdege et al [8] also found that NPS users often report substance use disorder symptoms, especially craving.

This community may have some other distinct characteristics. A minority of the sample reported that an NPS was the first drug that they had ever taken. Of those who ceased using NPS, majority found it either easy or very easy to stop. Most commonly, cessation was due to the side effects of NPS [17]. They also perceived internet forums as an important channel through which to communicate information on new drugs, and retailers reported monitoring forums to determine which drugs to stock in their store [8]. These users also tend to post online warnings based on first-hand experiences about the potential harms of the substances consumed and are willing to avoid harm to their peers. Orsolini et al [13] even stated that posting online the *on drug* experience report is arguably the *trait d’union* of all e-psychonauts, considering the intention behind using a substance is the most significant difference between a psychonaut and a typical drug user. O’Brien et al [11] also identified the role of e-psychonauts in disseminating emerging information about NPS-related harm and considered them well equipped to make a valuable contribution to NPS policy debates in general, and e-psychonauts are ideally placed to report on the effects of recent policy changes on NPS-related harms in particular.

Several authors have tried to engage cyber-psychonauts as research participants. Mdege et al [8] found difficulties in involving NPS users throughout the project due to a lack of willingness on the part of NPS users to be contacted in ways other than email. In addition, working with this population has inherent sampling problems: internet research participants are, by definition, a nonrandom and self-selecting sample, and it is very difficult to know the characteristics of the overall pool from which the sample is drawn [18,19].

Different authors believe that these internet communities are a huge opportunity for researchers. The qualitative analysis of how different groups interact with online communities may help to systematize and codify needs, values, and preferences that are relevant to the group [20]. In internet communities, researchers can simply recruit participants or even go further and engage drug users more fully in dialog [21]. Some authors even state that the lack of physical presence and separate physical settings all reduce researcher control and power, thereby potentially leading to a more balanced relationship between researchers and participants [22,23]. In any case, e-psychonauts are a hidden, hard-to-reach population that may have a significant influence on future drug trends.

Some authors even consider cyber-psychonauts to be ideally placed to become involved in the actual implementation of innovative responses to the increasing prominence of NPS markets, as it is difficult to imagine a more efficient method for the rapid dissemination of new information about things such as the adverse effects of new products to consumers [24].

The recent alarm related to the growth of the NPS market and the gradual shift from the street to the cyber-drug market may call for the implementation of preventive tools and practices tailored to these new drug users’ characteristics [13].

Finally, in their empirical and conceptual revision of the NPS research field, Mdege et al [8] concluded that there was a clear need to move beyond an expert-driven discourse on NPS and involve people who use NPS as active and valuable research collaborators and stakeholders instead of passive research participants.

It is clear then that this sentinel population might be difficult to reach and retain in a highly structured study protocol [5,8,18,21,25]. However, the collaboration of the Energy Control (EC) International Drug Checking Service might provide a critical opportunity for recruitment, offering a free chemical analysis of the substance they want to consume. This service is already being used by this population in the main internet communities of psychonauts, and it has a well-regarded institutional presence in most of them [26,27].

### Relevance and Goals of This Study

In summary, NPS pose a public health threat at different levels, and there is a lack of research on the effects of the emerging substances as well as on which ones are appearing now. In addition, the research conducted to date has been unable to cover important gaps, such as studying relevant sentinel populations of e-psychonauts using new technologies, involving them in the research, and obtaining confirmatory analytical data of the substances studied. Old methodologies repeatedly fail to reach the skyrocketing turnover pace of newer NPS in the e-market. By the time old trials recruit the necessary drug *X* study participants and engage them in the old trial machinery, drug *X* has already become obsolete and has been replaced by *Y* and—possibly—even *Z* drugs.

This study aims to bridge the abovementioned evidence gaps. To do so, a naturalistic, observational, and longitudinal design has been adopted, recruiting e-psychonauts to gather information on their characteristics and the substances that they might be using before their popularization. This has been possible thanks to the development of a new online ad hoc tool designed for this project: an online platform thought to enhance communication with the e-psychonauts and allow community building. In addition, subjective effects on these substances have been studied, allowing the participants to send samples to a partner laboratory with gas chromatography (GC)/mass spectrometry (MS) in Barcelona and administering drug effect questionnaires in the most resembling way possible to the drug laboratory studies.

This design has resulted in the first internet-based, multinational study on a key sentinel population with laboratory confirmation on the composition of the reported samples.

### Study Objectives and Hypothesis

The study has been designed to answer 3 main research questions:

1. Who are the people who first try the new substances when they emerge in the market?
2. What are the substances emerging right now and their subjective effects?
3. Is it possible to collect reliable data to answer these questions using a low-budget internet platform and the design used in this study?

The researchers’ initial hypotheses are as follows:

1. The population of e-psychonauts will be made up of functional and educated people who use drugs mainly in a recreational way.
2. During the study period, we will be able to identify a wide variety of different substances, some of which have never been reported before in the scientific literature or by the organizations aimed at controlling illicit drug supply.
3. The study design and implementation will attract enough research participants with sufficient commitment to provide valuable, reliable, and meaningful data to generate quality evidence.

## Methods

### Overview

This study aims to discover the characteristics of the e-psychonaut population and the effects of the NPS they use, with a longitudinal design and no control group. The study is conducted online, recruiting participants using an innovative and specifically developed platform as part of the study project: Global Research and Analysis of New Substances Project (GRASP).

The study has been designed and will be reported using the Checklist for Reporting Results of Internet E-Surveys [28] and the STROBE (Strengthening the Reporting of Observational Studies in Epidemiology) statement checklist for observational studies [29], with the support of the SPIRIT (Standard Protocol Items: Recommendations for Interventional Trials) statement checklist of 2013 [30].

The study protocol was submitted in October 2018 and was approved by the Clinical Research Ethics Committee (Parc de Salut Mar, Barcelona, Spain, ref. 2018/8283/I) in January 2019. The study was conducted according to the Declaration of Helsinki recommendations and the emerging recommendations for online research on sensible topics [21,25]. All data collected online on the participants were encrypted according to the European and Spanish data protection regulations (2016/679 European Parliament and 27/4/16 reglamento general de protección de datos [general law about data protection] Spanish Royal Decree).

### Study Setting

The study is conducted mainly though internet, using 3 main tools:

1. The specifically designed GRASP platform
2. The *Qualtrics* survey service licensed though Columbia University
3. The Google Suite platform as an email service to contact candidates and attend to the private questions and concerns of the study participants.

In addition, the samples were received through traditional mail in the EC Headquarters in Barcelona, where they were initially processed and identified. The samples were then transported to the EC laboratory at the Hospital del Mar Research Institute (IMIM), second floor, to be analyzed using the techniques described below. The research team worked at both the EC headquarters and the IMIM laboratories. During the COVID-related lockdown that was established in Spain in March 2020, the laboratory analysis was interrupted for 3 months.

The usability of the platform and the multiple automated processes, such as *sending an email with a specific link to a questionnaire*, and the logic pathways (adaptive questioning) and validation requirements used in the *Qualtrics* questionnaires were systematically tested by the research team. A checklist of all possible scenarios was devised, and they were all executed by a blind research team member and the principal investigator. Once errors were identified, they were corrected, and the process was repeated from the beginning. In addition, participants were encouraged to report any problems or ideas to improve the procedures, so changes could be implemented when needed during the study.

### Participants

All the participants were correctly and fully informed by writing (refer to the participant information sheet in Multimedia Appendix 1) and prompted to ask any questions by email. In that case, answers were provided until the candidate confirmed that they had no more questions and were satisfied with the information received. All participants indicated their agreement to participate and signed an informed consent (IC) form (Multimedia Appendix 2) that was sent to the project email address and checked by the principal investigator before inclusion. It was not possible for candidates or participants to answer any online questionnaires without previously receiving the specific link, which was sent by the research team only when the participant met the criteria to fill the questionnaire. Participants were asked to sign with their online usernames to further protect their physical identity, as recommended by Barratt et al [21,25]. Participants received no monetary compensation for their participation, but instead, they were offered the possibility to get the NPS they reported on analytically tested for free in the EC laboratory, located in the *IMIM*, Barcelona. The cost of this service is US $110 if contracted independently through the EC International Drug Checking Service.

This study included e-psychonauts, who have been defined as people with the following characteristics:

1. Previous experience with at least three NPS 12 months before the study inclusion
2. Activity on online communities where NPS consumption is discussed.

The inclusion and exclusion criteria for participants are provided in Textbox 1.

**Inclusion and exclusion criteria for participants.**

**Inclusion Criteria**

- Meeting the operational definition of e-psychonaut
- Being 18 years or older at the time of recruitment
- Self-reported plans of maintaining the use of new psychoactive substances for the following 18 months

**Exclusion Criteria**

- Difficulties in communicating in English
- Difficulties in using new technologies to participate in the study
- Potentially pregnant women
- Potential presence of severe psychopathological symptomatology

Note that there is no restriction on the geographical location of the participants, as the study will not collect such data to further protect the participants’ physical identity. Therefore, participants from around the globe could participate in the study. Both inclusion and exclusion criteria have been mainly assessed by direct self-reporting in an initial screening questionnaire (Q0), except the following:

1. Previous participation in forums has been assessed by self-report and exclusive advertisement of the study on these forums.
2. Difficulties in using new technologies have been assessed by the steps required to complete the screening process, such as sending an IC form in a particular format, registering to the platform, and following the instructions there to introduce themselves to the research team and other participants.
3. Difficulties in communicating in English and the presence of potential psychopathological impairments have also been evaluated by the principal investigator, assessing the answers to long and elaborate open questions in the screening questionnaire (Q0) and in the written introductions to the online platform.
4. The potential of being pregnant was assessed by indirect questioning using the same screening questionnaire (Q0) questionnaire.

### Recruitment

Recruitment ads were sent to the moderators of the selected online communities after establishing bilateral communication with them, mainly to ask permission and explain the goals of the project. To maximize interest in participating in the study, the only focus was on establishing rapport with community leaders, as if they share their interest in the study, they will be able to transfer it to the rest of the community [31]. Each community moderator posted the research ad in the most appropriate way in their community after discussing it with the research team. Research ads were posted on all communities during the summer of 2019 (refer to Multimedia Appendix 3 for details). The recruitment was designed to be sequential until the designed sample size was reached or the study reached its duration limit.

The operational definition for online communities of people who use NPS has been adapted from the study by Barratt [21]:

1. Surface websites with at least 5 years of existence
2. Presence of participation forums dedicated to discussing the use of NPS
3. At least weekly activity on the community forum
4. The use of pseudo-anonymity by community members to identify themselves
5. The presence of official and analytical drug checking services in the community.

When the study design was completed (October 2018), there were 4 communities meeting the previously stated criteria [21]:

1. *Bluelight* [32]: Established in 1997, bluelight is probably the most prominent community of people who take illicit drugs, with approximately 250,000 members. Within the community, there is a subdivision in which the use of NPS is exclusively discussed. The community is known for its commitment to promoting risk management and harm reduction strategies among its participants as well as its formidable contributions to similar research projects [28]. Registration is required to access the content.
2. *Reddit* [33]: Established in 2010, this subreddit community allows almost any type of discussion regarding NPS. The community has approximately 90,000 members, but it is part of a broader community of people who use illicit drugs (not only NPS), with over 700,000 members. Both of these are part of the global reddit community, where all types of topics are discussed. The platform does not require registration to access the content.
3. *Drugs-forum* [34]: Established in 2003, this community also seems to have approximately 250,000 members. Registration is required to access the content.
4. *DNstarsVIP*: Established after discussing about NPS sources was banned on the *reddit* community, *DNstars* is a strongly emerging community with approximately 2000 users. Registration is required to access the content.

The main communities that were assessed and excluded were as follows:

1. *Legal-highs forum*: excluded because of the lack of weekly interactions, technical website problems, and impossibility to contact community managers.
2. *Erowid*: excluded because of the lack of an active forum.
3. *Psychonaut wiki*: excluded because of the lack of an active forum.
4. *Tripsit*: excluded because of the lack of an active forum, although there was an internet relay chat–supported chatroom.
5. *Dimethyltriptamine-nexus:* excluded because of the lack of a specific NPS subsection.
6. *Ecstasy data*: excluded because of the lack of an active forum and the lack of a specific NPS subsection.
7. *Shroomery:* Excluded because of the lack of a specific NPS subsection.

To the best of the authors’ knowledge, the selected communities were the main ones at the moment when the selection occurred (October 2018), although it has to be acknowledged that this is a rapidly changing scenario; in a few years, this same process might produce different outcomes. At that moment, the authors were unable to find any contradicting information with that assumption. Soussan et al [35] referred to *bluelight*, drugs-forum, and legal-highs forum as the top 3 communities. However, they did not consider collaboration with drug checking organizations to be a relevant factor. Moreover, as stated above, these rankings are expected to change over relatively short periods [35].

The GRASP platform, which allowed for interaction among participants themselves and with the research team, was the main tool to promote participant engagement and minimize dropout rates.

### Sample Size

In the most recent review consulted by the authors, the sample sizes reached with web-based questionnaires in people who use illicit drugs ranged from 80 to 9867 [36]. The expected losses while filling these types of questionnaires are about 50% of the sample, but the authors have not found other online longitudinal studies including questionnaires like this one. According to the aforementioned review, the authors expected 80 candidates as the best possible estimate to achieve 40 final participants. Assuming that each participant takes one NPS every month during the duration of the study, the expected number of registered self-administration trials of NPS would be 480. As the study has been designed as exploratory, the sample size could not be determined based on the needs to perform specific statistical tests.

### Study Procedures and Timeline

The study’s internal timeline and workflow are graphically represented in Multimedia Appendix 4. The first recruitment effort consisted of online discussions with forum moderators and posting the institutional review board (IRB)–approved announcement for candidates (displayed in its entirety in Multimedia Appendix 3) in those forums. In the announcement, potential candidates were instructed to send an email from a secure and nonidentifiable address to the research team (admin@grasp.pw). Candidates were then informed more broadly about the study. Candidates were informed homogeneously by sending the IRB-approved *information for candidates’ sheet* to their email (the sheet used in this study can be found in Multimedia Appendix 1). The principal investigator then offered the candidates to answer any questions that might have arisen after reading the participant information sheet. When the participants had read and discussed the given information about the study with the principal investigator, they were asked to register on the GRASP platform and send the IRB-approved IC form, completed with the registered username to the study email. In Multimedia Appendix 2, the IC form is available for consultation. Finally, the candidates were asked to complete the screening questionnaire (Q0) and introduce themselves on the platform without providing information that might reveal their real-world identities. When all these processes were complete, the principal investigator checked the IC form, the screening questionnaire, and the platform introduction to assess if the participant met the inclusion or exclusion criteria. Candidates were then informed by email about the results of the assessment, thus either being rejected or accepted as participants.

Once participants were accepted, they could interact with other participants on the online platform and received detailed instructions on how to conduct the study. However, NPS sourcing and the effects of the substances included in the study were not allowed. In case of a severe protocol violation such as this one, participants were immediately removed from the study and their information was deleted. In case of minor protocol violations, participants were notified and given the opportunity, if applicable, to amend their noncompliant behavior.

The first mandatory step was to fill a sociodemographic and drug use history questionnaire (Q1). This questionnaire was available to each participant through a participant-specific link, which was sent by email once they were accepted. After that, participants were asked to fill the sample submission questionnaire (Q2), where information about the sample they intended to consume was asked. This questionnaire was then reviewed by the principal investigator and approved if the substance met the study criteria of being a new psychoactive substance. The samples that did not meet the inclusion criteria were not accepted, and the participant was notified by email. The sample submission questionnaire (Q2) was available to all accepted participants as a link on the platform. All the questionnaire answers were reviewed weekly by a member of the research team to communicate to the participant the acceptance of the sample and to mark them as valid or invalid data for later analysis.

If the sample was approved, the participant received a specifically generated sample code with the instructions to send a small amount of the sample (approximately 30 mg, usually below the psychoactive threshold) via traditional mail to the laboratory at the IMIM. The sample was analyzed there, and the result was sent back to the user, along with harm reduction advice when appropriate.

Meanwhile, the users could consume the substance whenever they decided, as the study was intended to be observational. However, most of the participants waited until they had the result of the laboratory analysis to proceed with the self-administration trial. The self-administration trial started with the users filling the drug effect baseline questionnaire (Q3a), and then, they consumed the reported substance and filled the drug effect questionnaire (Q3b) 24 hours after filling the baseline questionnaire (Q3a). The links to these questionnaires were available for all participants in the forum, and the veracity of the information was ensured by asking information only available to each participant, such as the sample code of the reported sample.

Study recruitment began in August 2019 and is still ongoing. In August 2020, the first participant concluded the 1-year follow-up.

### IRB-Approved Protocol Changes During the Study

The protocol has been subjected to amendments twice, both approved by the IRB of the institution (Clinical Research Ethics Committee-IMIM).

The first amendment, submitted in January 2019, reported the following changes in the protocol:

1. Minor changes in the study advertisement sheet, participant information sheet, and IC form
2. The assessment of inclusion criteria was no longer done by the community moderators and was entirely assessed by self-reporting on online questionnaires
3. An increase in the required sample quantity to be sent to the laboratory from 30 to 50 mg by default, accepting exceptions depending on the substance potency
4. Addition of a key measurement timepoint at baseline before ingesting the substance.

The second amendment, submitted in November 2019, reported the following changes in the protocol:

1. Unblinding of the research team to the participant behavior and participation
2. Addition of an optional timepoint for data collection in the reporting of the subjective effects of the reported substances
3. Extension of the duration of the study from 6 months to 1 year for each participant
4. Reduction of the required age for inclusion from 21 to 18 years.

### Outcomes

The domains and measurements used in the study are based on previous laboratory studies, to maximize consistency in methodology and to eventually develop a validation study for this methodology. Multiple studies have shown that web-based data collection and traditional methods (eg, paper and pencil) result in equivalent conclusions, demonstrating the validity and reliability of online data collection for research [37,38].

Certain studies have been used as model references to select the measured outcomes [39-52]. However, new outcomes regarding the subjective effects of psychoactive substances have been added to balance the amount of positive and negative effects reported. The order of the questions was kept the same to facilitate reports to those participants who completed the same questionnaire multiple times. However, 3 questions to assess validity were present in both the Q3a and Q3b. More information about study outcomes, including assessed domains, chosen measurements, metrics, and time points, can be found in Multimedia Appendix 5.

### Data Collection

#### Questionnaires

The study data were collected using a Columbia University Q*ualtrics* license, a well-known questionnaire platform compliant with the guidelines to store sensible information about the research participants. The screening questionnaire (Q0) and the sociodemographic questionnaire (Q1) collected self-reported information about the participants’ medical and drug history, psychosocial situation, and beliefs and behaviors related to drugs.

The subjective effects of drugs were assessed via visual analog scales, using the same parameters used in most laboratory studies to determine the subjective effects of drugs. The main outcome was the difference in mm from the drug effect questionnaire (Q3b) at 24 hours (referring to the peak experience) and the baseline questionnaire (Q3a).

Each questionnaire also had at least one validity entry to be filled by the researcher directly using the *Qualtrics* database. There were no automated consistency or completeness checks before the questionnaire was submitted, other than the validation criteria for certain questions. For example, the question *sample code reported* could not be submitted if the answer was not a 5-digit number.

The participants could go back through the questionnaire, but once submitted, they could not change their answers. A summary of the answers was not displayed either before or after submission. Two options were available to change the participants’ answers if they were incorrect according to the participant or not valid according to the researcher. If the change was small, the researcher could just edit the participant response in the *Qualtrics* database according to the correct response provided by the participant using email or the platform’s private messaging system. If the changes were relevant, the researcher could mark the questionnaire as invalid and provide another link to the participant.

Public links to copies of the used questionnaires can be found in the references cited below:

1. Q0, screening questionnaire [53]: contained a total of 37 questions
2. Q1, sociodemographic questionnaire [54]: contained a maximum of 351 questions, with an expected average per participant of 50, due to adaptive questioning and questionnaire logic
3. Q2, sample submission questionnaire [55]: contained a total of 21 questions
4. Q3a, baseline drug effect questionnaire [56]: contained a total of 12 questions
5. Q3b, drug effect questionnaire given 24 hours after drug administration [57]: contained a total of 39 questions
6. Q4, 1-year follow-up questionnaire [58]: contained a maximum of 351 questions, with an expected average per participant of 50, due to adaptive questioning and questionnaire logic.

The number of pages and items on each page were optimized automatically by *Qualtrics* software and varied according to the screen size used to answer. The possibility to answer the questionnaires comfortably from the smartphone was assessed as an essential by the research team.

#### Laboratory Analysis

Preliminary sample identification was performed by GC coupled to MS using an Agilent 7890B gas chromatograph coupled to a 5977A quadrupole mass spectrometer detector (Agilent). The gas chromatograph was fitted with a G4513A auto-sampler injector. Insert liners packed with salinized glasswool were used, and the injector and interface were operated at 280 °C. Samples were injected in split mode into a 0.25 mm film thickness (5% phenylmethylsilicone) column (HP-5MS, Agilent Technologies). Helium was used as the carrier gas at a flow rate of 1 mL/min. The oven temperature was initially maintained at 90 °C for 2 minutes and programmed to reach 320 °C at 20 °C/min. It was finally maintained at 320 °C for 9.5 minutes (total run time was 21.5 min). The mass spectrometer was operated in the electron impact ionization mode at 70 eV. To confirm the mass spectra, 4 libraries were used: the NIST/EPA/NIH Mass Spectral Library, Data Version: NIST 14; Searchable Mass Spectral Library Version 2.3 [59]; Searchable Mass Spectral Library Cayman Spectral Library [60]; and EC’s internal mass spectral library. Confirmation (when needed) was performed by liquid chromatography (LC) coupled to tandem MS (LC/MS/MS) using an Agilent 1100 series HPLC (high performance liquid chromatography) chromatograph (Agilent Technologies) and an Esquire 3000 plus mass spectrometer MRM (Bruker Daltonic GmbH). Chromatography was performed using a Poroshell 120 EC-C18 column (100 mm length×2.1 mm internal diameter; 2.7 mm particle size) at 30 °C. The mobile phases consisted of 1% formic acid and 1% formic acid in methanol. The following gradient elution was used: at time 0 minute, 15% B was changed to 90% B in 7 minutes, held for 1 minute, and changed back to the initial conditions in 1 minute. Before injection of the next sample, the column was re-equilibrated for 7 minutes. The flow rate was 0.35 mL/min. The electrospray source was operated in the positive ionization mode. Product ions that were obtained by collision-induced dissociation allowed the MS/MS to be operated in the multiple reaction monitoring mode. The dwell time was set at 0.25 seconds. The desolvation gas was nitrogen set at 365 °C and delivered at a flow rate of 9 L/min. The capillary voltage was 3.90 kV, and the collision gas was helium. The Bruker Compass Hystar system software Version 3.2-SR2 was used for instrument control and identification.

#### GRASP Forum

Secondary data about the participants’ discussions on the study platform were supported by a licensed *discourse* (Civilized Discourse Construction Kit, Inc) account and the software used to build the platform. *Qualtrics* (SAP Global Corporate Affairs) data were downloaded for analysis, which was conducted using the institutionally licensed Microsoft Excel from the IMIM and R (R Core Team), which is a free software environment for statistical computing and graphics that does not require a license.

### Data Management and Statistical Analysis

Data will be stored in *Qualtrics* software and will only be used according to the goals of the study described in the protocol. Most of the data will be entered directly by the participants, and a researcher will screen every questionnaire filled for consistency and ask the participants for clarifications in case of suspected errors in data entry or reporting. The principal investigator (MG) and 3 more researchers (GMF, XCM, and JGC) will have access to the complete data sets. Data on participant performance will be entered manually by one researcher in an internal Excel database. No personal information will be stored other than the *safe* email address asked in the study advertisement and the nickname the participant choses to use in the forum. This ensures the maintenance of pseudo-anonymity, as information about the online persona will be stored, but the link between the online identity and the real identity will not be impossible to establish with the collected information.

Data will be managed and processed using *Qualtrics* software and initially analyzed using Excel by the research team. At the same time, an independent statistician will use the same data from *Qualtrics* to perform a partially blind analysis using R. Only fully completed and valid questionnaires will be analyzed. The validity of the questionnaires will be assessed by a research team member based only on the consistency and completeness of the participants’ answers.

As it is an exploratory study, the data analysis procedures will be mainly descriptive statistics, to maximize an adequate visualization of the data collected within the minimum space. In addition, as there is no control group, statistical tests will be limited to potential comparisons to assess bias on the results, such as comparing data from participants who complete the study with participants who drop out or are excluded. However, no statistical corrections will be applied to adjust the representativeness of the sample, as this validation will be done by comparing the sociodemographics of the study sample with the characteristics of the population of psychonauts widely reported in the literature. Statistical tests might also be used to compare information from the initial questionnaire Q1 and the data from the follow-up questionnaire Q4. The nonparametric Wilcoxon signed-rank analysis will be used to perform a mean comparison of the quantitative data and the chi-square test for qualitative data. In addition, factor analysis will be attempted to study the relationship between all the visual analog scales used to assess the subjective drug effects.

The data analysis will focus on the outcomes of the participants who completed the study. Data entries containing evident errors or inconsistent information will be discarded, and the extent to which this might have impacted the results will be reported.

### Ethical Considerations

This study was approved by the IRB of IMIM on December 11, 2018, after the first submission in October 2018, when clarifications were asked and delivered in November 2018.

The study has been designed and is being executed according to the basic principles of rights and dignity of the human being, as stated in the Helsinki declaration, and this study complies with all the current regulations that apply, including institutional, local, national, and international regulations.

All information is being handled confidentially according to the organic Spanish law 15/1999 and the European regulation 2016/679. The IRB has always granted access to any study information required.

All participants will receive the participant information sheet and will be required to read and fill the IC form, which will be sent to admin@grasp.pw. The principal investigator will be responsible for reviewing all candidates’ IC forms and screening questionnaires for inclusion and exclusion criteria. All candidates and participants will have the opportunity to ask as many questions as needed before proceeding to any part of the study, both through the forum and through contacting the leading researcher email (admin@grasp.pw).

Participants will not be required to sign IC forms to protect their anonymity and avoid sharing data that could be used to track their physical identity. The identity that will be protected by the researchers will be the online one, as no other information relatable to the real identities will be given. This procedure is consistent with the methodology of previous studies [61].

The researchers will try their best to limit the influence of their interactions on the participants’ behavior, especially the ones targeted in the study. However, as recommended by previous research, the participants will be involved in discussing the study design and incentivized to share their opinions on how the study could be improved [8,21].

Protocol changes will be communicated to the participants through the online platform once they are approved.

A more extensive ethical analysis by principles can be found in Multimedia Appendix 6 [26,27,39-52,62-64].

### Dissemination Policy

All individual and collective data, as well as all the results derived from the study, will be strictly protected and will only be published with the authorization of the principal investigator and the affected participants. All relevant findings will be sent for publication in suitable journals and submitted for presentation at relevant scientific meetings. The funding organizations will have no role in the publication process. In addition, data without identifiable information will be shared with study participants after assessment and approval by the research team. Finally, all results published in the scientific literature will also be made available in lay language to the communities of origin of the participants. Authorships in the publications will be determined by the amount of scientific and academic contributions of the members of the research team, including external collaborators. There are no plans to make the data sets publicly available.

## Results

The research protocol was approved by the IRB of the *IMIM* on December 11, 2018. Data collection started in August 2019 and was still ongoing when the protocol was submitted (September 2020), finalizing in October 2020.

Data analysis began in November 2020, and it is still ongoing. The authors expect to submit the first manuscript with preliminary results by the end of 2021.

From a total of 182 screened candidates, only 17 (9.3%) completed at least one self-administration trial, resulting in a total number of 64 self-administration trials. From these, 40 different substances were analyzed.

## Discussion

It is possible to conduct an IRB-approved study using this new methodology and collect the expected data. However, the meaning and usefulness of these data are still unknown.

## Appendix

Multimedia Appendix 1 Participant information sheet.

Multimedia Appendix 2 Informed consent form.

Multimedia Appendix 3 Announcement for candidates.

Multimedia Appendix 4 Summary of study procedures.

Multimedia Appendix 5 Main study outcomes.

Multimedia Appendix 6 Ethical analysis by principles.

## Abbreviations

EC: Energy Control
EC–ABD: Energy Control–Associació Benestar i Desenvolupament
GC: gas chromatography
GRASP: Global Research and Analysis of New Substances Project
HPLC: high performance liquid chromatography
IMIM: Hospital del Mar Research Institute
IC: informed consent
IRB: institutional review board
LC: liquid chromatography
MS: mass spectrometry
NPS: new psychoactive substances
SPIRIT: Standard Protocol Items: Recommendations for Interventional Trials
STROBE: Strengthening the Reporting of Observational Studies in Epidemiology

## References

[1] Higgins K, O’Neill N, O’Hara L, Jordan J, McCann M, O’Neill T, Clarke M, O’Neill T, Campbell A (2019). Evidence for public health on novel psychoactive substance use: a mixed-methods study. Public Health Res.

[2] Schifano F, Napoletano F, Chiappini S, Guirguis A, Corkery JM, Bonaccorso S, Ricciardi A, Scherbaum N, Vento A (2019). New/emerging psychoactive substances and associated psychopathological consequences – corrigendum. Psychol Med.

[3] Corazza O, Assi S, Simonato P, Corkery J, Bersani FS, Demetrovics Z, Stair J, Fergus S, Pezzolesi C, Pasinetti M, Deluca P, Drummond C, Davey Z, Blaszko U, Moskalewicz J, Mervo B, Furia LD, Farre M, Flesland L, Pisarska A, Shapiro H, Siemann H, Skutle A, Sferrazza E, Torrens M, Sambola F, van der Kreeft P, Scherbaum N, Schifano F (2013). Promoting innovation and excellence to face the rapid diffusion of novel psychoactive substances in the EU: the outcomes of the ReDNet project. Hum Psychopharmacol.

[4] World Drug Report 2017. United Nations Office on Drugs and Crime.

[5] Meader N, Mdege N, McCambridge J (2018). The public health evidence-base on novel psychoactive substance use: scoping review with narrative synthesis of selected bodies of evidence. J Public Health (Oxf).

[6] Davidson C (2012). New psychoactive substances. Prog Neuropsychopharmacol Biol Psychiatry.

[7] European Monitoring Centre for Drugs and Drug Addiction (2018). Monitoring Drug Use in Recreational Settings Across Europe: Conceptual Challenges and Methodological Innovations, Technical Report.

[8] Mdege ND, Meader N, Lloyd C, Parrott S, McCambridge J (2017). The novel psychoactive substances in the UK project: empirical and conceptual review work to produce research recommendations. Public Health Res.

[9] Weaver MF, Hopper JA, Gunderson EW (2015). Designer drugs 2015: assessment and management. Addict Sci Clin Pract.

[10] Wood DM, Heyerdahl F, Yates CB, Dines AM, Giraudon I, Hovda KE, Dargan PI (2014). The European Drug Emergencies Network (Euro-DEN). Clin Toxicol (Phila).

[11] O'Brien K, Chatwin C, Jenkins C, Measham F (2014). New psychoactive substances and British drug policy: a view from the cyber-psychonauts. Drugs Educ Prev Policy.

[12] Corkery JM, Orsolini L, Papanti D, Schifano F (2017). From concept(ion) to life after death/the grave: The 'natural' history and life cycle(s) of novel psychoactive substances (NPS). Hum Psychopharmacol.

[13] Orsolini L, Papanti GD, Francesconi G, Schifano F (2015). Mind navigators of chemicals' experimenters? A web-based description of e-psychonauts. Cyberpsychol Behav Soc Netw.

[14] Newcombe R, Johnson M (1999). Psychonautics: A model and method for exploring the subjective effects of psychoactive drugs. Proceedings of the Club Health 2000 – First International Conference on Nightlife and Substance Use.

[15] Tackett-Gibson M (2009). Constructions of risk and harm in online discussions of ketamine use. Addict Res Theory.

[16] Winstock A, Mitcheson L, Ramsey J, Davies S, Puchnarewicz M, Marsden J (2011). Mephedrone: use, subjective effects and health risks. Addiction.

[17] Fletcher E, Tasker S, Easton P, Denvir L (2016). Improving the help and support provided to people who take new psychoactive substances ('legal highs'). J Public Health (Oxf).

[18] Andrews D, Nonnecke B, Preece J (2003). Electronic survey methodology: a case study in reaching hard-to-involve internet users. Int J Hum Comput Interact.

[19] Eysenbach G, Wyatt J (2002). Using the internet for surveys and health research. J Med Internet Res.

[20] Tonks A, Smith R (1996). Information in practice. Br Med J.

[21] Barratt M, Lenton S (2010). Beyond recruitment? Participatory online research with people who use drugs. Int J Internet Res Ethics.

[22] Hewson C, Joinson A, McKenna K, Postmes T, Reips UD (2009). Gathering data on the internet: qualitative approaches and possibilities for mixed methods research. Oxford Handbook of Internet Psychology.

[23] Illingworth N (2017). The internet matters: exploring the use of the internet as a research tool. Sociol Res Online.

[24] Seddon T (2014). Drug policy and global regulatory capitalism: the case of new psychoactive substances (NPS). Int J Drug Policy.

[25] Barratt M (2011). Discussing illicit drugs in public internet forums: visibility, stigma, and pseudonymity. Proceedings of the 5th International Conference on Communities and Technologies.

[26] Brunt TM, Nagy C, Bücheli A, Martins D, Ugarte M, Beduwe C, Vilamala MV (2017). Drug testing in Europe: monitoring results of the Trans European Drug Information (TEDI) project. Drug Test Anal.

[27] Measham FC (2019). Drug safety testing, disposals and dealing in an English field: exploring the operational and behavioural outcomes of the UK's first onsite 'drug checking' service. Int J Drug Policy.

[28] Eysenbach G (2004). Improving the quality of web surveys: the Checklist for Reporting Results of Internet E-Surveys (CHERRIES). J Med Internet Res.

[29] Cuschieri S (2019). The STROBE guidelines. Saudi J Anaesth.

[30] Chan A, Tetzlaff J, Altman D, Laupacis A, Gøtzsche P, Krleža-Jerić K, Hróbjartsson A, Mann H, Dickersin K, Berlin J, Doré C, Parulekar W, Summerskill W, Groves T, Schulz K, Sox H, Rockhold F, Rennie D, Moher D (2013). Spirit 2013 statement: defining standard protocol items for clinical trials. Chinese J Evidence-Based Med.

[31] Ip EJ, Barnett MJ, Tenerowicz MJ, Perry PJ (2010). The touro 12-step: a systematic guide to optimizing survey research with online discussion boards. J Med Internet Res.

[32] Bluelight.org.

[33] Research chemicals discussion - strictly no sourcing.

[34] Drugs-Forum.

[35] Soussan C, Kjellgren A (2014). Harm reduction and knowledge exchange-a qualitative analysis of drug-related internet discussion forums. Harm Reduct J.

[36] Miller PG, Sønderlund AL (2010). Using the internet to research hidden populations of illicit drug users: a review. Addiction.

[37] McCabe SE (2016). Comparison of web and mail surveys in collecting illicit drug use data: a randomized experiment. J Drug Educ.

[38] Buchanan T, Smith JL (1999). Research on the Internet: validation of a World-Wide Web mediated personality scale. Behav Res Methods Instrum Comput.

[39] Martin W, Sloan J, Sapira J, Jasinski D (1971). Physiologic, subjective, and behavioral effects of amphetamine, methamphetamine, ephedrine, phenmetrazine, and methylphenidate in man. Clin Pharmacol Ther.

[40] Folstein MF, Luria R (1973). Reliability, validity, and clinical application of the Visual Analogue Mood Scale. Psychol Med.

[41] Bozarth MA (1987). Methods of Assessing the Reinforcing Properties of Abused Drugs.

[42] Riba J, Valle M, Urbano G, Yritia M, Morte A, Barbanoj MJ (2003). Human pharmacology of ayahuasca: subjective and cardiovascular effects, monoamine metabolite excretion, and pharmacokinetics. J Pharmacol Exp Ther.

[43] Kleinloog D, Roozen F, De Winter W, Freijer J, Van Gerven J (2014). Profiling the subjective effects of Δ⁹-tetrahydrocannabinol using visual analogue scales. Int J Methods Psychiatr Res.

[44] Papaseit E, Farré M, Pérez-Mañá C, Torrens M, Ventura M, Pujadas M, de la Torre R, González D (2018). Acute pharmacological effects of 2C-B in humans: an observational study. Front Pharmacol.

[45] González D, Torrens M, Farré M (2015). Acute effects of the novel psychoactive drug 2C-B on emotions. Biomed Res Int.

[46] Haney M, Ward AS, Comer SD, Foltin RW, Fischman MW (1999). Abstinence symptoms following oral THC administration to humans. Psychopharmacology (Berl).

[47] Bouso JC, Pedrero-Pérez EJ, Gandy S, Alcázar-Córcoles MÁ (2016). Measuring the subjective: revisiting the psychometric properties of three rating scales that assess the acute effects of hallucinogens. Hum Psychopharmacol.

[48] Hart CL, Ward AS, Haney M, Nasser J, Foltin RW (2003). Methamphetamine attenuates disruptions in performance and mood during simulated night-shift work. Psychopharmacology (Berl).

[49] Cami J, Farré M, Mas M, Roset PN, Poudevida S, Mas A, San L, de la Torre R (2000). Human pharmacology of 3,4-methylenedioxymethamphetamine ("ecstasy"): psychomotor performance and subjective effects. J Clin Psychopharmacol.

[50] Farré M, de la Torre R, Llorente M, Lamas X, Ugena B, Segura J, Camí J (1993). Alcohol and cocaine interactions in humans. J Pharmacol Exp Ther.

[51] Farré M, Terán MT, Roset PN, Mas M, Torrens M, Camí J (1998). Abuse liability of flunitrazepam among methadone-maintained patients. Psychopharmacology (Berl).

[52] Hart C, Ward A, Haney M, Foltin R, Fischman M (2001). Methamphetamine self-administration by humans. Psychopharmacology (Berl).

[53] Q0 Screening questionnaire.

[54] Q1 Sociodemographic and drug use questionnaire.

[55] Q2 Sample characteristics.

[56] Q3a Experience report baseline.

[57] Q3b Drug effects assessment.

[58] Q4 Follow up questionnaire.

[59] SWGDRUG mass spectral library.

[60] Cayman Chemical.

[61] Barratt MJ, Ferris JA, Lenton S (2014). Hidden populations, online purposive sampling, and external validity. Field Methods.

[62] Fry C, Treloar C, Maher L (2005). Ethical challenges and responses in harm reduction research: promoting applied communitarian ethics. Drug Alcohol Rev.

[63] Karamouzian M, Dohoo C, Forsting S, McNeil R, Kerr T, Lysyshyn M (2018). Evaluation of a fentanyl drug checking service for clients of a supervised injection facility, Vancouver, Canada. Harm Reduct J.

[64] Saleemi S, Pennybaker SJ, Wooldridge M, Johnson MW (2017). Who is 'Molly'? MDMA adulterants by product name and the impact of harm-reduction services at raves. J Psychopharmacol.

