# Supplementary material for: Self-reported Subjective Effects of Analytically Confirmed New Psychoactive Substances Consumed by e-Psychonauts: Protocol for a Longitudinal Study Using a New Internet-Based Methodology
Source: JMIR Res Protoc. 2021 Jul 2;10(7):e24433. doi: 10.2196/24433 (PMC8285746; doi:10.2196/24433)
Supplement: Multimedia Appendix 1 [file resprot_v10i7e24433_app1.doc]

# Annex 1: Participant information Sheet

TITLE OF THE STUDY: Validation and application of a new online methodology to study the effects of new psychoactive substances

SHORTENED TITLE: : GRASP (Global research and analysis of substances project)

PROTOCOL VERSION AND CODE: 4th version; May 27, 2019; 2018/8283/I

PRINCIPAL INVESTIGATOR: Marc Grifell Guàrdia

RESEARCH CENTER: Hospital del mar research institute (IMIM) in collaboration with Energy Control (EC), Universitat Autònoma de Barcelona (UAB) and Columbia University (CU)

**Purpose of Study:**

The purpose of this research project is to study the subjective effects of new psychoactive substances and the characteristics of a population of committed participants in the main online communities of drug users.

You are confirming that you are not currently seeking treatment for your drug use, and if you are a woman that you are not pregnant. If you are interested in treatment for your drug use, please let us know and we can help you find it.

This study is funded by research funds from Hospital del Mar research institute (IMIM),

Energy Control (EC) and Columbia University (CU).

**Importance of the study**:

Information regarding new psychoactive substances is insufficient. Online surveys and self-reported data offer now timely information but sadly not reliable enough, due to the impossibility to confirm the actual substances consumed and asses the conditions in which they are consumed. Information from clinical trials, in the rare case it exists, takes too long to reach both the scientific community and the communities of drug users who want to make informed decisions.

This project pretends to fill the evidence gap between this two types of research in new psychoactive substances. To do so, a new methodology of study will be implemented with a threefold purpose:

To study the population of committed members of the main online communities of drug users

To characterize the subjective effects of the new psychoactive substances emerging during the period of study

To try this new methodology by comparing the results of the reported effects of traditional drugs with the existing database of laboratory studies with traditional substances

**Voluntary participation:**

Participation in this study is voluntary, and you may refuse to participate or stop participating at any time without loss of benefits to which you are otherwise entitled. You will be informed of any new findings or risks that arise that may affect your willingness to continue in this study. If you decide not to participate in this study or to withdraw at any time, this will not affect your present or future enrollment in other studies conducted by these same institutions or researchers. The investigator may also decide that your participation should be discontinued, if he/she thinks that this is better for you.

**Alternative to Participating in This Study**

This is not a treatment or intervention study. Information being collected is for research purposes only. The alternative to participating would be simply not to participate.

**Study Procedures**

If you agree to participate, you will fill a baseline form assessing your sociodemographic characteristics, drug use experience and medical history. This may involve questions about your mental health, physical health, drug use, sexual activity, legal history, and any problems you might be having.

Although we prefer that you answer all of the questions, you do not have to answer any questions that make you feel uncomfortable.

Once you have agreed to participate and signed the consent form, you will have access to an online platform where you will be able to discuss with other participants or the investigators any concern that you might have during the study. Participation regarding the study design and the study results will also be encouraged there. Experiences regarding the effects of the studied samples will not be allowed as discussion topic to avoid contamination among participants.

At any point that you intend to consume a substance during the period of study, you will be asked to fill a very short form informing which substance do you intend to use and if you want to include it in the study. In case the episode of drug use is included in the study you will be asked to fill an extensive report regarding the context of use and the subjective effects of the substance. Also, you will be asked to send a sample for chemical analysis performed either by GC/MS (gas cromathography / mass spectrometry) and HPLC (high pressure liquid cromathography). The sample will be analyzed for free and you will receive a report within a few days. Although it is recommended to wait for the report before ingesting the substance, this decision will be yours, as this is not an intervention study and we only intend to study a naturally occurring phenomena. The research team will be able to deny the free analysis of the substance after the short form is submitted and before the sample is sent (and the questionnaire filled). This measure is only intended to prevent the flooding of the analysis service by the same type of sample or under the suspicion of fraudulent use, that will be discussed with the user.

After one year of initiating your participation in the study, this will formally end, and participants will be expected to fill another extensive questionnaire about their drug use and personal situation at that moment. If permission is granted by the ethics committee and funding is available, participants might be given the opportunity to continue with the study for more time, although this possibility is not granted.

**Risks If you participate in the study:**

Because all of the potential risks of the studied behaviors to an unborn baby, women should not be in this study if they are pregnant, breastfeeding, or possibly pregnant. If you think you might be pregnant at any point, please tell the investigator.

The following risks may be involved with the study procedures.

Some people have found the questionnaires to be uncomfortable and/or tiring. Some of the questions we may ask could be embarrassing. You can refuse to answer any questions.

The study might be time consuming, with an expected dedication of 4-6 hours a month.

As the study is a naturalistic study, it will not substantially modify the risks of the behaviors the participants usually engage in.

**Confidentiality**

The treatment, communication and cession of the data gathered in the study will be according to the new European data protection regulation, Regulation (EU) 2016/679 of the European Parliament and the council of April 27, 2016 (RGPD).

Additionally, we will never ask you for your name, address or any information that can be related to your physical identity.

We will need you to provide us with an email address that you will need to confirm and use for the study proceedings. This e-mail address can be yours or specifically set up for this study to protect your identity.

Once you enter the study you will be assigned a study code and you will choose a study username.

You will be able to exert your right to the destruction of all information relative to your participation at any point during the study, which would also imply your withdrawal from it. You will only need to contact the research team through the information provided below to exert that right.

Specific collected data without identifiable information might be shared with other researchers. This data would not contain either your email or the chosen username.

**Final remarks**:

If you have any questions, please ask. The investigators will answer to the best of their abilities any questions you may have now or in the future about the study procedures.

You should contact the Principal Investigator, Dr. Marc Grifell Guàrida at [admin@grasp.pw](mailto:admin@grasp.pw) if you have any questions.

The hospital del mar research institute research ethics committee has approved the recruitment of participants for these studies. A research ethics committee is a committee that protects the rights of participants in research studies. If you have any questions about your rights as a research participant or any complaints, you may contact the committee at +34 93 316 06 79 from 9am to 2pm (Spanish time).

Please save a copy of this document for your personal use.
