# Supplementary material for: Self-reported Subjective Effects of Analytically Confirmed New Psychoactive Substances Consumed by e-Psychonauts: Protocol for a Longitudinal Study Using a New Internet-Based Methodology
Source: JMIR Res Protoc. 2021 Jul 2;10(7):e24433. doi: 10.2196/24433 (PMC8285746; doi:10.2196/24433)
Supplement: Multimedia Appendix 2 [file resprot_v10i7e24433_app2.doc]

# Annex 2. Informed consent.

**Title of the study**: Validation and application of a new online methodology to study the effects of new psychoactive substances.

**Shortened title:** GRASP (Global research and analysis of substances project).

I have discussed this study with _________________ to my satisfaction.

To the best of my knowledge, I am not pregnant.

I understand my participation is voluntary.

The investigator has explained to me the risks derived from consuming new psychoactive substances with unknown effects, I am aware of these risks and here I testify that I have decided to consume NPS at my own responsability independently of my particpation in the study.

Signing this form does not waive any of my legal rights.

I may choose not to participate or to discontinue my participation at any time without penalty or loss of benefits to which I am otherwise entitled. I voluntarily agree to participate in the research study described above.

Participant username ________________________________________________________

I have discussed the proposed research with this participant and, in my opinion: this participant understands the benefits, risks, and alternatives (including non-participation) and is capable of freely consenting to participate in this research.

Investigator_________________________________________________________________
