# Supplementary material for: Self-reported Subjective Effects of Analytically Confirmed New Psychoactive Substances Consumed by e-Psychonauts: Protocol for a Longitudinal Study Using a New Internet-Based Methodology
Source: JMIR Res Protoc. 2021 Jul 2;10(7):e24433. doi: 10.2196/24433 (PMC8285746; doi:10.2196/24433)
Supplement: Multimedia Appendix 3 [file resprot_v10i7e24433_app3.doc]

# Annex 3 Announcement for candidates

Research project needs experienced NPS users to collaborate

The principal investigator of this study is [Marc Grifell Guàrdia](https://www.americanscientist.org/author/marc_grifell), from Hospital del Mar research Institute (IMIM) and Autonomous University of Barcelona (UAB).  The research team is comprised of leading figures in the field like [Dr. Carl Hart](https://www.youtube.com/watch?v=C9HMifCoSko) from Columbia University and [Dra. Mireia Ventura](https://www.researchgate.net/profile/Mireia_Vilamala)  from Energy Control.

The goal is to provide fast and reliable information about subjective effects of new drugs when they emerge in internet communities.

We believe that there is a gap between no-analytically confirmed trip reports or surveys and clinical trials with healthy volunteers. We want to implement a new methodology combining the speed of the self-reported information with the reliability of clinical trials. For this reason, we have asked forum moderators to contact committed and experienced members of their communities to help us develop this project.

If you decide to participate, you will be invited to a private forum where free drug checking services will be provided to all participants (GC/MS and HPLC). There, you will asked to systematically report the effects of the NPS you have decided to test. Also, we hope you will engage in a discussion with the research team to design the second phase of this study.

We believe that people like you can make a difference in the development of scientific knowledge if provided with the necessary tools. With your help, we hope to make self-experimentation a valid source of scientific knowledge. We would like that the reliable, balanced and timely information produced in this study will be able to shape drug policy.

Please send an email to [admin@grasp.pw](mailto:admin@grasp.pw) if you are interested. We want to protect both your virtual and physical identity, so the only identifiable information will be the email address from which you send the message. Do not add any other information. Please make sure it’s anonymous and you check it regularly. We’ll send a time-sensitive invitation there.
