# Supplementary material for: Self-reported Subjective Effects of Analytically Confirmed New Psychoactive Substances Consumed by e-Psychonauts: Protocol for a Longitudinal Study Using a New Internet-Based Methodology
Source: JMIR Res Protoc. 2021 Jul 2;10(7):e24433. doi: 10.2196/24433 (PMC8285746; doi:10.2196/24433)
Supplement: Multimedia Appendix 4 [file resprot_v10i7e24433_app4.doc]

# Annex 4: Study procedures summaries

**
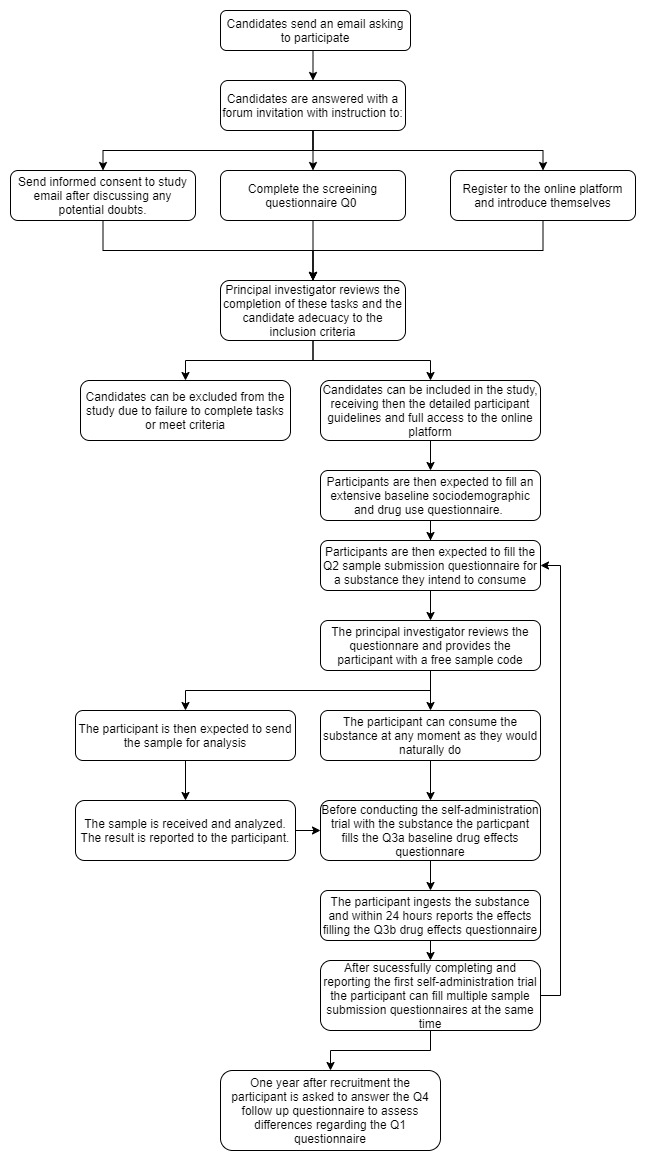
**

Figure 1 Participant expected procedures during the study


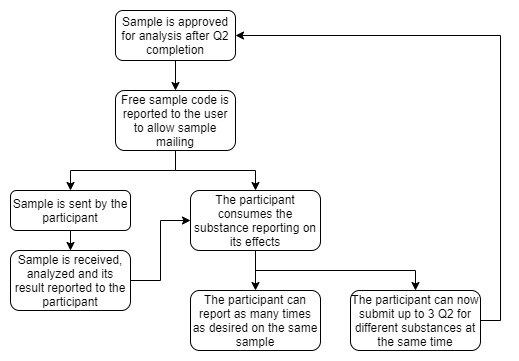


Figure 2 Sample processes flow-chart
