# Supplementary material for: Self-reported Subjective Effects of Analytically Confirmed New Psychoactive Substances Consumed by e-Psychonauts: Protocol for a Longitudinal Study Using a New Internet-Based Methodology
Source: JMIR Res Protoc. 2021 Jul 2;10(7):e24433. doi: 10.2196/24433 (PMC8285746; doi:10.2196/24433)
Supplement: Multimedia Appendix 5 [file resprot_v10i7e24433_app5.doc]

# Annex 5: Study main outcomes

## **Table 1.** Sociodemographics

| **Domain** | **Measurement** | **Variable characteristics** | **Timepoints** | **Analysis metric** |
| --- | --- | --- | --- | --- |
| Age | Absolute number of years | Numeric | Q0 | Mean, Standard deviation |
| Sex | Self-perceived sex | Three categories (M/F/other) | Q1, Q4 | Percentatge |
| Geographic location | Self-reported country | Free text | Q1, Q4 | Percentatge |
| Education | Completed studies | 7 fixed categories | Q1, Q4 | Percentatge |
| Income | Self-reported last year annual income | Numeric | Q1, Q4 | Mean, median, Q1, Q3 and range (Box plot) |

## **Table 2.** Medical history:

| **Domain** | **Measurement** | **Variable characteristics** | **Timepoints** | **Analysis metric** | |
| --- | --- | --- | --- | --- | --- |
| Medical or psychiatric history | Self-reported | Open text in structured battery for common symptoms, diseases and milestones such as hospitalizations | Q1,Q4 | Perfentage;  Difference Q1 to Q4 |  |

## **Table 3.** Patterns of drug use (for each substance or substance category)

| **Domain** | **Measurement** | **Variable characteristics** | **Timepoints** | **Analysis metric** |
| --- | --- | --- | --- | --- |
| Age of first consumption | Self reported age | Numeric | Q1 | Mean, median, Q1, Q3 and range (Box plot) |
| Route of administration | Self reported | 7 fixed categories | Q1, Q4 | Perfentage;  Difference Q1 to Q4 |
| Frecuency of drug use | Self reported use in time-period | 4 fixed categories (last month, last, year, more than one year ago, never) | Q1,Q4 | Perfentage;  Difference Q1 to Q4 |
| Drug related problems | Self reported for the 11 SUD criteria | Dicothomic (yes/no) | Q1, Q4 | Perfentage;  Difference Q1 to Q4 |

**Table 4.** Self administration trial (SAT) report

| **Domain** | **Measurement** | **Categories** | **Timepoints** | **Analysis metric** |
| --- | --- | --- | --- | --- |
| Route of administration | Self-reported | 7 fixed categories | Q3a | Perfentage; |
| Ingested dose | Self-reported | Numerical (mg) or approximation | Q3a | Mean, median, Q1, Q3 and range (Box plot) |
| Duration and intensity of effects | Self reported | VAS of intensity of effects at each hour for 12 hours | Q3b | Mean and Standard deviation for each timepoint |
| Craving at comedown | VARS mm | Numerical | Q3b | Mean, median, Q1, Q3 and range (Box plot) |
| Subjective drug effects | Visual Analog scales (see next section for detail) | Numerical | Q3a Q3b | Difference from baseline. (Q3b-Q3a) |
| Ingested substance | GC/MS; LC/MS | Categorical, list of NPS | Depending on when the sample is sent and duration of analysis | Percentage, difference from expectation, |

## Subjective effects assessed using visual analog scales (Q3a and Q3b)

| Good drug effect | High | Stimulated | Euphoric | Energetic | |
| --- | --- | --- | --- | --- | --- |
| Happy | Talkative | Calm | Self confident | Friendly |  |
| Alert | Social | Able to focus | Well coordinated | Self-accepting |  |
| Understanding of others feelings | Physically comfortable | Interested in self-reflection | Pleasurable body sensations | Forgiving yourself or others |  |
| Able to face my current life challenges | Please mark the minimum value (validity item) | Feel presence of numinous force, higher power, god | Remembering important moments of my life | Insightful about personal or occupational concerns |  |
| Able to hear others | Well treated, gentle | With improved sense of humor | Aware of my body | Emotionally open |  |
| Trusting of others | Creative | In control | Body lightness | Motivated |  |
| Attentive | Able to “let go” | Smiling | Honest | Positive |  |
| Patient | Sexy | Awe, amazement |  |  |  |
| Bad drug effect | Anxious | Sad or depressed | Sedated | Confused |  |
| Incompetent | Fearful | Mentally slow | Forgetful | Dizzy |  |
| Nauseous or like vomiting | Limb heaviness | Stomach pain | Tired | Miserable |  |
| Headache | Blurred vision | Irritable | Suicidal | Dry mouth |  |
| Jittery | Clumsy | Tremor | Muscle pain | Chills |  |
| Sweating | Aggressive | Numbness | Chest pain | Urge to move |  |
| Please mark the maximum value (validity item) | Experiencing difficulties to find words | Heart pounding or beating faster | Urge or craving to consume more | Hot or cold flushes |  |
| Itchiness |  |  |  |  |  |
| Any effect | Active | Changes in lights | Changes in colors | Passive |  |
| Increased sexual desire | Change in skin sensitivity | Different surroundings | Changes in distances | Changes in hearing |  |
| Please mark between the middle and the maximum (validity item | Hallucinations-seeing animals, things, insects, or people | Hallucinations-seeing of lights or spots | Different or changed unreal body feeling | Like reflecting about my childhood |  |
