# Supplementary material for: Self-reported Subjective Effects of Analytically Confirmed New Psychoactive Substances Consumed by e-Psychonauts: Protocol for a Longitudinal Study Using a New Internet-Based Methodology
Source: JMIR Res Protoc. 2021 Jul 2;10(7):e24433. doi: 10.2196/24433 (PMC8285746; doi:10.2196/24433)
Supplement: Multimedia Appendix 6 [file resprot_v10i7e24433_app6.doc]

# Annex 6: Ethical analysis by principles

## Non-maleficence

No harms to the study participants are expected. The cost for the participants will be their time answering the questionnaires and the shipment expenses related to sending the samples to the study laboratory. Their privacy will be kept using different overlapping strategies, such as log-in records, use of personal usernames and passwords for different research members with different access to data and encryption using PGP, among others. Also, the participants will only relate with the researchers with their virtual identities, which will also be protected. This has been described as pseudo-anonymous participation [25]. Finally, when the study ends, the only information linking the virtual identity of the participants (the email address) will be removed from the study databases.

The interactions in the GRASP platform will be monitored to avoid interventions that can lead to increased or harmful drug use. This will be done by the weekly review of all content and the eventual elimination of messages or participants that might cause harm to others. The lack of physical presence of the researchers and the absence of a controlled environment similar to the traditional clinical trials setting clearly reduces subjects perception of a hierarchical relationship between researchers and them, something that in previous research has been reported as positive, leading to a more symmetrical relationship between both roles [22,23].

## Respect for persons and autonomy

As commented above, the increased symmetry between researchers and participants will secure their autonomy and dignity. Also, active efforts will be made to enroll participants in a pro-active attitude towards all aspects of the study, including having access to the data and manuscripts before publication so their opinion and voice will be heard. This approach has been described by some authors as necessary for all human research in the 21st century [62].

## Individual subject benefits

The study participants will have the opportunity to use free drug checking services during their participation int he study. This is an established harm reduction practices that has shown to improve user’s safety and, in some cases, save their lives [26, 27, 63, 64]. Thanks to free drug checking, the study participants will have the opportunity to discard adulterated samples or mislabeled samples containing other substances than expected, increasing the safety of a risky behavior they were already engaged in [27].

## Justice:

All researchers except the principal investigator will be blinded to the gender, race, or origin data of the participants, minimizing the potential for discrimination. However, the recruitment will happen globally, and it is likely that the recruited sample will not be representative of the global diversity in its multiple layers and intersections. The researchers have not been able to avoid this limitation and hope that the results of the study will help to better understand the profile of the population to better inform future studies in that regard.
